# Physical activity interventions for women with metastatic breast cancer: a systematic review of published and ongoing randomised controlled trials

**DOI:** 10.1007/s11764-025-01917-y

**Published:** 2025-11-11

**Authors:** Louise H. Hall, Sophie M. C. Green, Zainab Haider, Abigail Fisher, Natalie King, Rebecca J. Beeken, Samuel G. Smith

**Affiliations:** 1 Leeds Institute of Health Sciences, University of Leeds, Leeds, LS2 9NL UK; 2 Institute of Epidemiology and Health Care, University College London, London, WC1E 7HB UK

**Keywords:** Physical activity, Metastatic breast cancer, Systematic review, Randomised controlled trials, Interventions, Exercise

## Abstract

**Purpose:**

We systematically reviewed published and ongoing physical activity (PA) trials in women with metastatic breast cancer (MBC). We examined (i) the effectiveness of PA interventions and identified (ii) the type of interventions being evaluated, (iii) how they are delivered and (iv) their theoretical basis.

**Methods:**

Seven databases and two trial registries were searched in August 2024 for randomised controlled trials, testing any PA intervention in people with MBC, reporting a PA outcome. The Joanna Briggs Institute (JBI) handbook was followed, including quality assessment using the JBI Critical Appraisal Checklist for RCTs. Data were summarised narratively. Intervention details were extracted using the TIDieR framework.

**Results:**

One thousand six hundred eighty-seven records were screened and 96 assessed for eligibility. Twenty-eight reports were included (13 full reports, 4 protocols, 11 trial registries). Sample sizes ranged from 21 to 357 participants. Twenty-one out of 28 reports were phase II, pilot, or feasibility trials. Most interventions did not cover all types of recommended PA. The methodological quality of studies was moderate. Intervention adherence was moderate to high (≥ 50% in 10 studies). Among studies reporting on safety (9), only one recorded any serious events (two events) related to the intervention. Evidence indicates that PA can improve fatigue, health-related QoL, physical fitness, and functioning over the short and medium term (≤ 6 months).

**Conclusions:**

Physical activity is safe, well adhered to, and improves physical function and QoL in MBC. Future trials could clarify the optimal PA type, duration, delivery mode, and long-term effectiveness.

**Implications for Cancer Survivors:**

Women with MBC should be supported by healthcare professionals to be active.

**Supplementary Information:**

The online version contains supplementary material available at https://doi.org/10.1007/s11764-025-01917-y.

## Introduction

Treatment advances in metastatic breast cancer (MBC) have improved survival across all disease sub-types [1]. There were an estimated 167,518 prevalent cases of metastatic breast cancer (MBC) in the USA in 2020, a figure projected to rise to 246,194 by 2030 [2]. A quarter of this growing population will live over 5 years following diagnosis [3]. The medical and productivity costs of MBC are estimated to be $63.4B in 2015, increasing 140% to $152.4B by 2030 [2]. These costs are expected to be higher for younger and midlife women [4].

Maintaining functional quality of life (fQoL) is a priority for patients, but women currently undergo multiple lines of treatment, causing a wide range of short- and long-term physical and psychological consequences including reduced physical function, fatigue, pain, and distress [5–10]. Women experiencing severe symptoms are more likely to cease treatment early, which may impact survival [11]. While medical intervention may be required for some of these physical and psychological sequelae of treatment, effective self-management of mild to moderate symptoms could improve patient well-being and clinical outcomes.

Definitive evidence shows physical activity (PA) can improve fQoL in early stage breast cancer [12], and it is recommended in major guidelines (e.g. American Society of Clinical Oncology) [13, 14]. Although women with MBC are often excluded from PA trials due to safety concerns and historically poor prognosis, PA is considered safe [15, 16], including for those with bone metastases when modified by a trained professional [17–19]. Furthermore, observational evidence suggests PA could improve fQoL in women with MBC [20]. Improving fQoL should be prioritised, as across a range of disease sites and stages higher fQoL is associated with lower emergency admissions and hospitalisations [21], reduced sick leave [22, 23], and in MBC potentially longer survival [24–26].

Existing systematic reviews of PA interventions have included trials enrolling people with metastatic cancer across all disease sites, but few trials enrolled MBC patients [27–29]. While some PA interventions may be applicable to all disease sites, specific modifications may be required for MBC [30], and generalisations from other cancer sites should not be made without evidence of safety, efficacy, and feasibility in this patient group. A systematic synthesis of physical activity trials specifically in MBC is needed to inform the development of future intervention strategies. It could also provide a single resource of trials within this field for healthcare professionals (HCPs) wanting to implement PA in their clinical practice. We aimed to systematically synthesise the literature on published and ongoing PA trials in women with MBC. Our objectives were to examine (i) the effectiveness of physical activity interventions for increasing physical activity in this population and to identify (ii) the types of physical activity interventions being evaluated, (iii) how they are delivered, and (iv) the theoretical basis for them.

## Method

The review was conducted following the Joanna Briggs Institute’s Manual for Evidence Synthesis [31] and reported using the Preferred Reporting Items for Systematic Reviews and Meta-Analyses (PRISMA:2020) [32] (Supplementary File 1). It was pre-registered on PROSPERO (CRD42023462994).

### Search strategy

Searches for completed randomised controlled trials (RCTs) and published protocols of ongoing trials were conducted on 16th August 2024 in MEDLINE, Cochrane, CINAHL, PsycINFO, Web of Science, Scopus, and Embase. Ongoing trials were searched for on clinicaltrials.gov and ICTRP on 20th August 2024. Databases were searched from inception to the search date. Keywords were identified from known papers on similar topics. An information specialist (NK) created our search strategy for MEDLINE and reviewed our search strategies for all other databases. See Supplementary File 2 for search terms for MEDLINE, and the Open Science Framework (OSF) [>https://doi.org/10.17605/OSF.IO/2SW76] for search terms for all databases. Keywords and MESH terms were searched for ((metastatic OR advanced) breast cancer) AND (physical activity OR exercise OR weight training OR resistance training), with filters for randomised controlled trials applied.

### Eligibility criteria

Studies were included if they used an RCT design (including pilot and feasibility trials), were available in English, and met the following PICO criteria: Population: adults (aged 18+) with any type of advanced or MBC; Intervention and Context: any type of physical activity intervention delivered in any suitable setting; Comparator: usual care or a comparable intervention or any other suitable comparator; Outcome: measure of physical activity. Additional outcomes of interest included quality of life, fatigue, intervention adherence, and adverse events. Full eligibility criteria can be found on the OSF.

### Study screening and selection

Completed trials and published protocols were downloaded into Endnote and duplicates removed, before uploading onto Rayyan for screening. Titles and abstracts were independently screened in duplicate by RB, SS, LH. Full texts were independently screened in duplicate by SS and LH. Discrepancies were arbitrated by RB and SG. Forward citation searching and hand searching reference lists of included studies were conducted. Ongoing trials were downloaded into Microsoft Excel and duplicates removed before titles, abstracts, and full texts/registry entries were independently screened in duplicate by RB and LH, with discrepancies arbitrated by SS.

### Assessment of methodological quality

Results papers were independently assessed for methodological quality using the Joanna Briggs Institute Critical Appraisal Checklist for RCTs [33] in duplicate by RB, AF, and SS, arbitrated by SG. As the majority of studies were pilot and feasibility trials, they were appraised at the study level rather than the outcome level. Phase III trials were assessed for the primary outcome only.

### Data extraction

Data was extracted in duplicate by LH and SS (arbitrated by SG and ZH) in Microsoft Excel. The form collected data on study design, country, sample size, key clinical eligibility criteria, locations of metastases, disease sub-types, relevant treatment information, participant demographics, socio-economic variables, outcome measures and timepoints, patient-reported outcomes assessed, biomarkers assessed, effect sizes of primary (physical activity) and secondary outcomes of interest, intervention adherence, recruitment rate, retention, and adverse events (number and type). Intervention details were extracted using the template for intervention description and replication (TIDieR) framework [34]. Intervention adherence was categorised into low (< 50%) and medium/high (≥ 50%) based initially on a review of older adults [35], but increased (from 30%) due to the younger population under study in our review, who may be more physically able to adhere.

### Data synthesis

We undertook a narrative synthesis structured around the type of report (results, protocol, registry), study characteristics, types of outcome measures, intervention characteristics, and study findings. Summaries of the interventions are tabulated using the extracted data, and key information is described in text. Commonalities and differences between the overall populations of studies are identified and described. No meta-analysis was planned, as initial searches indicated high heterogeneity of content, context, and outcome measures.

LH and SS categorised the interventions as containing or not containing the following types of exercise: (1) aerobic; (2) resistance/strength training; (3) flexibility, stretching, mobility; (4) balance, functional; (5) other. These categories were based on WHO exercise recommendations [36], Harvard Medical School descriptions of important exercise types [37], a Taxonomy of PA interventions in Older Adults [38], and a systematic review that categorised types of PA interventions [39].

## Results

**Fig. 1:**
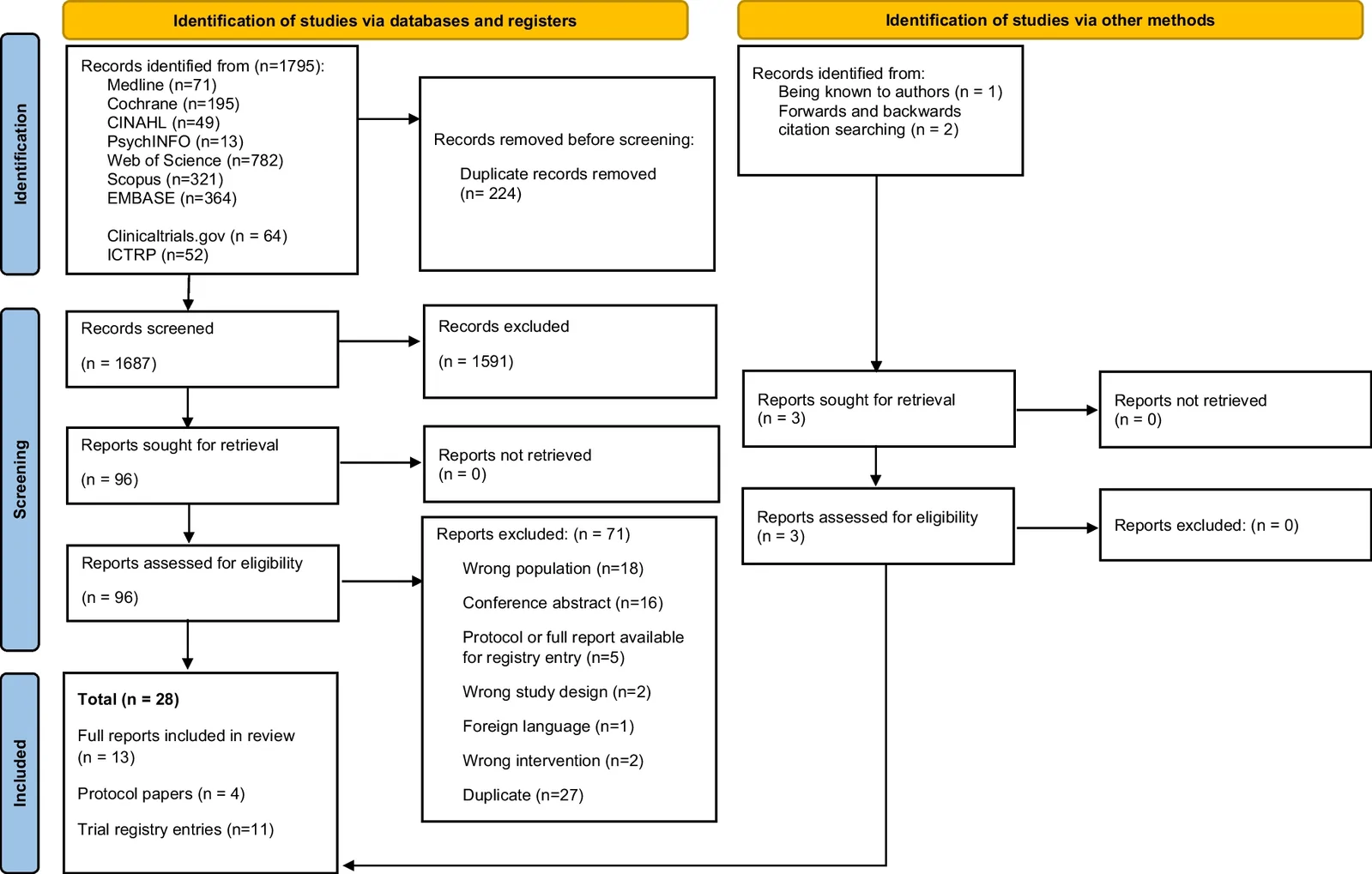
PRISMA flow chart

The flow of full reports, protocol papers, and trial registry entries is shown in Fig. 1. The database searches yielded 1795 records. After removing duplicates, 1687 records were screened and 96 were assessed for eligibility. Seventy-one records were excluded, leaving 25 reports for inclusion. After reviewing records known to the authors and completing backwards and forwards citation searching, an additional 3 reports were included. In total, 28 reports were included, including 13 full reports [40–52], 4 protocol papers [53–56], and 11 trial registry entries [57–67] (Table 1).

**Table 1:** Summary of characteristics in full reports, protocols, and trial registries

Authors/date	Country	Design	Sample size	Follow-up	Key eligibility criteria	Intervention/comparator	Primary outcome(s)	Participant characteristics	Findings	Safety
Full reports										
Carson, 2021 [40]	USA	Pilot, RCT (2:1)	63	Daily diaries, 8 weeks	MBC. Excluded ECOG ≥ 3 or KPS < 60, current engagement in yoga practice 1 or more days per week	Yoga vs. support group	Daily pain (0–9 self-report)	Mean age: 57.5; 74.5% White; 58.3% bone metastases; 14.6% brain metastases	No intervention x time interaction, suggesting no effect on daily pain (*p* = 0.275)	Not reported
Headley, 2004 [41]	USA	Pilot, quasi-RCT	38	12 weeks	MBC, scheduled to initiate outpatient chemotherapy, performance status ≤ 2, ability to sit on straight backed chair for 30 min. Excluded if receiving high dose chemotherapy or receiving only hormonal therapy	Seated exercise vs. usual care	Fatigue (FACIT-F)	51 years, 84.4% White	Intervention group had less decline in scores on fatigue subscale (*p* = 0.0078)	No safety events related to intervention
Hiensch, 2024 [51]	Germany, Netherlands, Poland, Spain, Sweden, Australia	Phase III, RCT	357	3, 6, 9 months	MBC, ECOG ≤ 2. Excluded unstable bone metastases, untreated brain metastases, and current engagement in intense exercise training	Multimodal: aerobic, resistance and balance + physical activity trackers vs. usual care + physical activity tracker + exercise advice	Fatigue (EORTC QLQ FA12) and health-related QoL (EORTC QLQ C30)	99.4% female; 55.4 years; treatment: endocrine therapy (52.3%); targeted therapy (57.1%); chemotherapy (25.5%); bone-modifying agent (46.5%). Metastases locations: bone (67.2%); lung (26.6); liver (34.7%); lymph node (38.7%). Triple negative (9.8%), HER2positive (23.2%), HER2negative/HR positive (59.7%)	Recruitment rate = 42%, Attrition at 6 months = 18.5% Exercise group had sig. improvements in HRQoL at 6 months (ES = 0.33, *p* = 0.0003, BGD = 4.8 (95% CI 2.2–7.4), lower physical fatigue (ES = 0.22, *p* = 0.27, BGD = 5.3, 95%CI = 10 to 0.6). Sig. improvements seen for physical fitness and various QLQ-C30 subscales	2 exercise-related SAEs unrelated to bone mets (wrist fracture; sacral stress fracture). 80 AEs including pain (51.3%), dizziness (12.5%), muscle soreness and/or cramps (11.3%), fatigue (8.9%); requiring modifications to the exercise programme
Ligibel, 2016 [42]	USA	Phase not described, RCT (1:1)	101	16 weeks	MBC or locally advanced disease; ECOG performance status 0 or 1; baseline performance of ≤ 150 min weekly recreational physical activity. Excluded women with untreated brain metastases	Aerobic exercise vs. wait-list control	Physical function: EORTC QLQ C30 and Bruce Ramp Treadmill Test	Median age: 49 years; 94% White; treatment: 53% endocrine therapy, 38.7% chemotherapy, 36.7% biologic therapy, 3% no treatment; 66% visceral metastatic disease	Non-significant improvement in physical function using EORTC QLQ C30 (*p* = 0.23) Non-significant increase in exercise duration using Bruce Ramp Treadmill Test (*p* = 0.35) Intervention participants increased their minutes of exercise by a mean of 62.4 ± 102.8 min per week versus 46.0 ± 154.3 min per week among controls (*p* = 0.17)	No safety events occurred
Oh, 2014 [43]	Australia	Phase II (feasibility), RCT	27	5 and 10 weeks	MBC; ECOG 0–2. Excluded women with brain metastases and already practicing Qigong/meditation once a week in last 6 months	Qigong vs. meditation	Adherence to programme (in person, home practice) and adverse events	57.4 years; 78.6% (intervention) 92.3% (control) White	63% completed study, 80% adhered to intervention, unknown proportion completed home practice 3–4 days/week for 15–30 min per day	No safety events occurred
Phillips, 2024 [52]	USA	Pilot/feasibility RCT	49	12 weeks	MBC, low levels of physical activity. Excluded untreated brain metastases	App-based PA promotion to promote walking and behaviour change + FitBit + weekly coaching calls vs. healthy lifestyle attention control	Feasibility: ≥ 80% completion rate (# complete 12-week assessment/# randomised); Adherence: % days wearing FitBit; acceptability: satisfaction	54.8 years; 85.7% White, 14.3% non-White (6.1% Black, 2.0% Asian pacific islander, 6.1% other or unknown). Hispanic (*n* = 5, 10.2%). Current treatment: 55.1% chemotherapy; 10.2% radiation therapy; 47.9% immunotherapy; 12.5% targeted therapy; 53.1% hormone therapy;	Completion rate = 98% Intervention group wore the Fitbit 92.7% of the time, met their step goal 53.4%, set a step goal 87.4%, used the app 94.1% of study days. ITT showed significant effect favouring intervention group on self-efficacy (*p* = 0.03), PA goal setting (*p* < 0.01). Trends (*d* = 0.025 to 0.45) for improvements on physical activity, (daily steps, activity counts, MVPA minutes, smaller declines in total PA and light PA, smaller increases in sedentary time)	1 death. No other adverse events were reported
Porter, 2019 [44]	USA	Pilot, RCT (2:1)	65	3 and 6 months	MBC. Excluded women if ECOG 3 +, KPS < 60; currently practicing yoga 1 + day/week	Yoga vs. social support	Acceptability (Client Satisfaction Questionnaire); feasibility (session attendance and assessment completion)	57.3 years; White (74.6%); treatment: radiotherapy (67.7%), surgery (24.6%), chemotherapy ongoing (64.6%)	82% indicated high satisfaction with intervention; 65% attended ≥ 4 sessions Intervention group walked a greater distance at post intervention and 6 months (6-minute walk test)	No safety events related to intervention
Schmitz, 2023 [45]	USA	Pilot/feasibility, RCT with partial crossover	42	3 months	MBC. Excluded women if ECOG > 2	mHealth vs. waiting list control	Acceptability (% agreeing to participate); feasibility (% interacting with platform at least 30/90 days)	53.4 years; White (90.5%)	51% consent rate for those known to be eligible; 64.9% logged in 30 of the 90 days; adherence declined each month after weekly calls; no BGD in sit-stand test	No safety events occurred
Schmitz, 2021 [46]	USA	Pilot/feasibility, RCT with partial crossover	21	3 months	MBC, ECOG ≤ 3	mHealth vs. waiting list control	Feasibility (proportion consenting, interacting with platform and participating in at least one month of programme) Acceptability (proportion of eligible patients agreeing to participate)	60 years; 94% White Treatment: no anti-cancer treatment (11.8%); systemic infusion therapy (70.6%); hormone therapy (64.7%); CDK 4/6 inhibitors (41.2%)	Feasibility: 48.3% (40.3/90 logged into Nurse AMIE. 13/17 (76%) who actively participated were still using at 1 month) Acceptability: 68% Signal of efficacy: no group x time interaction for pain, fatigue, distress or sleep	No additional healthcare visits incurred as a result of the intervention
Scott, 2018 [47]	USA	Pilot/feasibility RCT	65	3 months	MBC, ECOG 0 or 1, performance of < 150 min of moderate-intensity exercise per week	Supervised aerobic training vs. supervised stretching	Feasibility (loss to follow-up and attendance)	54 years Treatment: current therapy (chemotherapy (57%); endocrine therapy (48%); radiotherapy (5%); prior lines of therapy (0 = 12%; 1 = 40%; 2 = 31%; ≥ 3 = 35%)	Loss to follow-up (3%); mean attendance (63% ± 30%); permanent discontinuation of aerobic training (27%); 12% of sessions required a dose reduction. No BGD on functional capacity (6-minute walk test, sit-stand test)	Aerobic training group: 36% stopped a session early due to non-serious health event. 73% experienced at least 1 non-serious adverse event. No serious adverse events observed. Attention control group: 7 non-serious adverse events
Sheean, 2021 [48]	USA	Pilot/feasibility crossover RCT	40	12 weeks	MBC; ECOG 0–1, no change in anti-neoplastic therapies (30 days), no severe pain (≥ grade 3 NCI CTCAE), currently non-compliant with ACS nutrition and physical activity guidelines	Multimodal diet and physical activity vs. wait-list control	Feasibility (100% of recruitment goal) Adherence (> 75% telephone sessions and 2/3 exercise sessions) Efficacy (trends in improvement of QoL) Safety events	55 years, non-Hispanic White (91.4%); Current treatment: endocrine (45.2%); chemotherapy (25.8%); immunotherapies (71.0%)	Attendance: 86% (93% in person and 84% telephone sessions) Significant improvements in Fact G (*p* = 0.003) with improvements in physical, emotional and functional well-being; Increases in handgrip strength (*p* = 0.003) and time spent in physical activity (*p* < 0.001)	No adverse events related to the intervention; three women (2 intervention, 1 wait-list control) developed new symptoms/disease progression
Vadiraja, 2017 [49]	India	Phase not described, RCT	91	3 months	MBC; 30–70 years; ambulatory > 50% of time Excluded women with brain mets and who underwent chemotherapy	Yoga vs. supportive therapy and education	Not described	50.5 years	Improvements in fatigue severity, frequency, interference and diurnal variation (*p* < 0.0001)	Not reported
Yee, 2019 [50]	Australia	Pilot/feasibility RCT	14	8, 16 weeks	MBC; ECOG 0–2. Excluded women who regularly participated in physical activity	Supervised resistance training and unsupervised walking vs. usual care	Feasibility (recruitment, retention, adherence, safety)	62.2 years	Recruitment rate = 93%; retention rate = 100% [intervention]/83% [usual care]. 100% attendance to 16 supervised exercised sessions; all completed at prescribed intensity and volume. 2/8 (25%) women adhered to the walking program; compliance to walking intensity = 71%. Improvements shown in fatigue (FACIT-F ES = 1.92), pain (ES = 1.61), quality of life (physical, role, emotional, social function), and physical assessments	No safety events occurred
Protocols										
Delrieu, 2020 [53]	France	Phase III, RCT	244	HRQoL = 6, 12, 18, 36, 48 weeks, month 18 Other = months 3, 6, 12, 18	MBC with at least one positive hormone receptor (HR+) and HER2−	Walking (app and tracker) vs. physical activity recommendation	Time to deterioration of global health status and fatigue (EORTC QLQ C30)	N/A	N/A	N/A
Hart, 2018 [54]	Australia	Pilot/feasibility RCT	40	13 weeks	Stable osteolytic bone metastases in vervial, thoracic, and/or lumbar vertebrae; have not engaged in regular exercise in previous 3 months	Multimodal (resistance, aerobic and flexibility) focusing on spinal isometric training vs. usual care	Feasibility: recruitment, completion, safety, programme adherence and compliance. FACIT	N/A	N/A	N/A
Kirkham, 2021 [55]	Canada	Phase II, RCT	50	Pre cycle 1, pre cycle 3, post cycle 6 and 2 years	MBC, starting IV chemotherapy, ECOG ≤ 2	Cycling and dietary change during chemotherapy vs. Usual care PLUS one-time phone consultation with a registered dietitian and exercise physiologist vs. usual care plus one nutrition and exercise advice phone call	Change is lesion size (CT scan)	N/A	N/A	N/A
Meier-Girard, 2020 [56]	Switzerland	Phase III, RCT	196	8, 14, and 20 weeks	MBC, FACIT-F score < 34, ECOG ≤ 1	Eurythmy + CoordiFit vs. CoordiFit only	FACIT-F subscale	N/A	N/A	N/A
Trial registries										
Ballinger, 2022 [57]	USA	Phase II, RCT	100	8 and 16 weeks	MBC, ECOG 0–2, excluded: women receiving cytotoxic chemotherapy in past 12 months, active untreated brain metastases	Multimodal (cardiovascular, resistance + balance/stretching) vs. usual care plus leaflet	Cardiorespiratory fitness (minutes on treadmill)	N/A	N/A	N/A
Borquist, 2024 [67]	Denmark	Feasibility RCT	21	1 year, 1.5 years	MBC, BMI ≥ 25, receive first-line endocrine-based therapy, ECOG 0–1	Aerobic, resistance, stretching vs	Recruitment rate	N/A	N/A	N/A
Chamberlin, 2012 [58]	USA	Pilot/feasibility RCT	4	2, 3, 4, 5, 6 months	MBC, Karnofsky performance status ≥ 80%; excluded women with untreated CNS disease	Walking vs. usual care	Physical activity/steps (exercise logs and pedometer)	N/A	N/A	N/A
Crane, 2024 [66]	USA	Phase III, RCT	260	3, 6, 12 months	ER+ and/or PR+, HER2 metastatic or locally advanced BC; excluded unstable bone metastases, unstable cardiac disease, oxygen dependant, not consistently meeting exercise guidelines in past 3 months	Exercise (aerobic + resistance) vs. exercise + fasting vs. fasting only vs attention control (bi-weekly remote health education)	Fatigue (EORTC QLQ C30)	N/A	N/A	N/A
Dal Molin, 2022 [65]	Brazil	Phase not described, RCT	68	2, 4, 6 months		App + SmartWatch nutrition and physical activity vs. App only	Quality of life (FACT-B)	N/A	N/A	N/A
Devoto, 2014 [59]	Australia	Phase not described, RCT	48	Weeks 1, 4, 6, and 14–21 days post week 6	MBC, experiencing fatigue, ECOG 0–2; excluded: recent or anticipated change to systemic therapy, bone metastases with lytic metastatic lesions involving long bones	Multimodal (Aerobic and resistance) vs. active control (undefined)	Fatigue (FACIT-F)	N/A	N/A	N/A
Harvie, 2015 [60]	UK	Phase II, RCT	134 (planned) 68 (final)	Day 1 of each chemotherapy cycle, plus every 9–12 weeks during treatment. 15 months follow-up		Resistance training + diet vs. resistance training alone	Progression-free survival	N/A	N/A	N/A
Liu, 2020 [61]	Australia	Pilot/feasibility, RCT	20	12 and 18 weeks		Behavioural counselling vs. attention control (physical activity recommendation, handout, FitBit and diary)	Physical activity (Actigraph Accelerometer and IPAQ = SF)	N/A	N/A	N/A
Lozano-Lozano, 2022 [62]	Spain	Phase not described, RCT	58	14 weeks		Aerobic vs. usual care	Functional capacity (6-minute walk test) and Quality of life (EORTC QLQ C30)	N/A	N/A	N/A
Reeves, 2022 [63]	Australia	Phase III, RCT	200	3, 6, 9, 12 months		Aerobic, resistance exercises + diet vs. usual care + encouragement to access services	Physical function (EORTC QLQ C30) and whole body lean mass (DXA body scan)	N/A	N/A	N/A
Somayeh Nazari, 2023 [64]	Iran	Phase not described, RCT	28	8 weeks		Resistance training vs. no intervention	Liver enzymes ALT, AST and ALP	N/A	N/A	N/A

*RCT* randomised controlled trial, *MBC* metastatic breast cancer, *ECOG* Eastern Cooperative Oncology Group, *KPS* Karnofsky Performance Scale, *FACIT-F* functional assessment of chronic illness therapy-fatigue, *EORTC QLQ C30* European Organization for the Research and Treatment of Cancer Quality of Life Questionnaire, *CDK* cyclin-dependent kinase, *NCI CTCAE* National Cancer Institute Common Terminology Criteria for Adverse Events, *ACS* American Cancer Society, *QoL* quality of life, *HRQoL* health-related quality of life, *IPAQ-SF* International Physical Activity Questionnaire-Short Form, *DXA* dual-energy X-ray absorptiometry, *ALT* alanine transaminase, *AST* aspartate transaminase, *ALP* alkaline phosphate, *ES* effect size, *BGD* between group difference

### Full reports

#### Quality assessment

Table 2 summarises the methodological quality of full reports. True randomisation was used in all except two studies, from the same authors, which lacked detail about the randomisation procedure [45, 46]. Those allocating participants to groups were concealed from allocation in eight studies [40, 44, 47–52], but it was unclear in the remaining five [41–43, 45, 46]. As expected for behavioural interventions, no study blinded participants or intervention deliverers to treatment allocation. Outcome assessors were not blinded to treatment allocation in two studies [50, 51], and this was unclear in another six [41, 43, 45–47, 49].

**Table 2:**
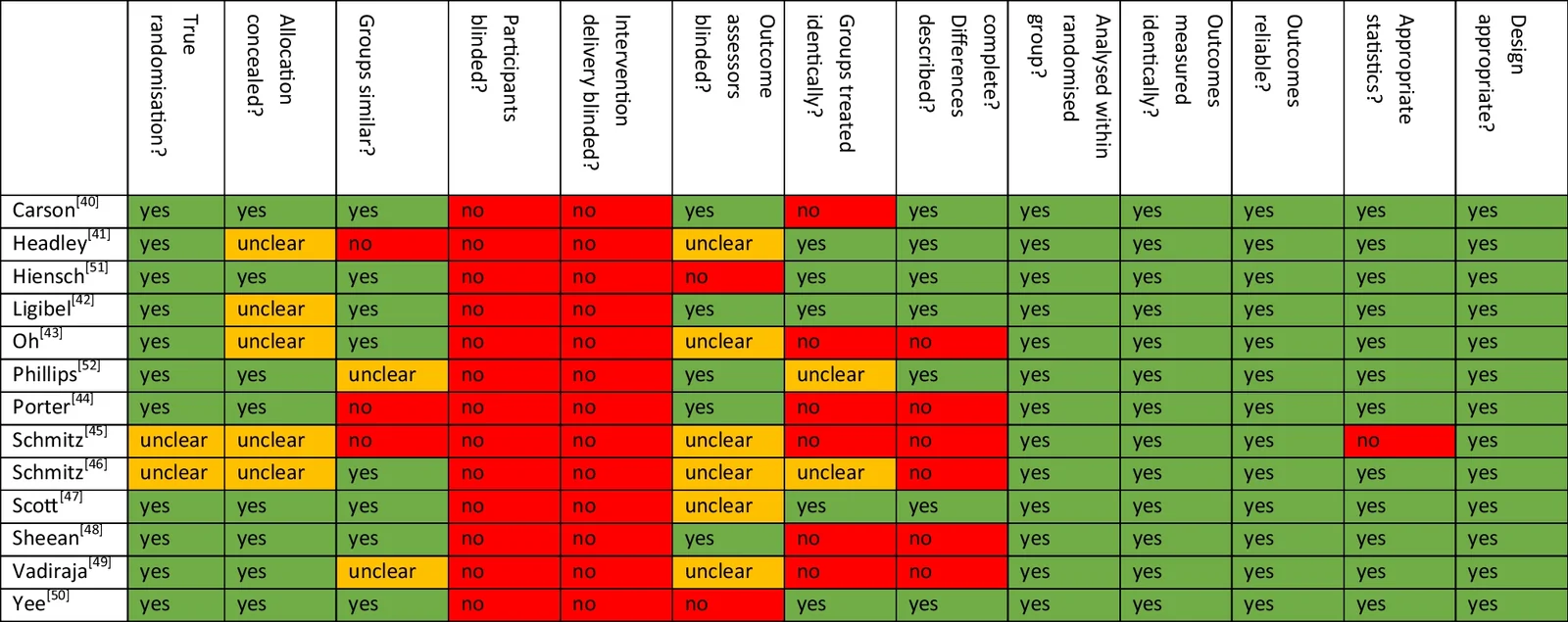
Summary of quality assessments using the Joanna Briggs Institute Critical Appraisal Checklist for Randomized Controlled Trials

Baseline differences existed between groups in three studies [41, 44, 45]; including differences in education level [41, 44], marital [41], and employment statuses [45]. The intervention and control groups were treated differently in six studies [40, 43–45, 48, 49]. For example, offering the control group support unavailable to the intervention group, such as enhanced usual care [43] or additional communication [48].

Differences between groups regarding follow-up (e.g. descriptions of loss to follow-up (LTF), reasons for LTF, impact of incomplete follow-up) were inadequately described in six studies [43–46, 48, 49]. All studies had reliable outcomes that were measured identically across groups, used appropriate designs and analysed participants in the groups they were randomised. One study was considered to not have used appropriate statistical analysis [45], which involved a complex crossover design but conducted separate comparisons for the immediate and delayed receipt of the intervention with the control.

#### Characteristics of studies

Most (61.5%) full reports originated in the USA [40–42, 44–48, 52], with representation also from Australia [43, 50], India [49], and various European countries [51]. Three were full reports of phase III trials [42, 49, 51], with the remainder being phase II [42] or pilot/feasibility studies (69.2%) [41, 41, 44–48, 50, 52]. Nearly all used a parallel group RCT design, with a minority using an RCT with full or partial crossover [45, 46, 48]. One trial reportedly used a quasi-experimental design, but it was unclear how the allocation method was not random [41]. Sample sizes ranged from 21 [46] to 357 [51] (median = 49).

Participant characteristics were not consistently reported, with 9/13 reporting on ethnicity [40–46, 48, 52], and 9/13 reporting any socio-economic variable [40, 41, 43–45, 48, 50–52]. Of these, participant samples were primarily White (74.5–94.0%) with the average age ranging from 49 [42] to 62.2 [50] years. Of the seven studies reporting employment status, the percentage of participants working full- or part-time ranged from 28.6% [44] to 61.2% [52]. Eight papers reported on recruitment rates, which ranged from 21.1% [52] to 93.0% [50] (mean = 49.8%).

Disease sub-type, treatment information, and location of metastases were not always reported. Where they were, bone metastases (29–67.2% of participants, reported in 4 studies [40, 48, 50, 51]) and visceral metastases (up to 71% reported in 6 studies [40, 42, 47, 48, 50, 51]) were common. Seven studies included participants with an Eastern Cooperative Oncology Group (ECOG) rating of ≤ 2 [40, 41, 43–45, 50, 51], one included those with ECOG of ≤ 3 [46], and three with an ECOG of ≤ 1 [42, 47, 48].

Primary outcome measures were self-reported pain [40], fatigue [41], quality of life [51], feasibility, and acceptability [43–48, 50, 52]. One trial had co-primary outcomes of physical function assessed by the EORTC QLQ C30 and the Bruce Ramp Treadmill Test [42]. One trial did not specify their primary outcome [49]. Two reports listed safety as a primary outcome [48, 50]. Follow-up ranged from 8 weeks [40] to 9 months [51]. Four full reports used a wait-list comparator [42, 45, 46, 48], two used usual care [41, 50], and seven [40, 43, 44, 47, 49, 51, 52] used a variety of approaches to enhanced usual care (e.g. attention controls [47, 52], support groups [40, 44]).

#### Intervention characteristics according to the TIDieR checklist

Table 3 summarises the intervention characteristics according to the TIDieR checklist. Supplementary File 3 summarises the full data extraction of the TIDieR components. Three full reports referred to a theory or theoretical framework when describing the intervention [41, 48, 52]: two were based on social cognitive theory [48, 52], but one of these was not justified or described in detail [48]. One study reported using Roy’s Adaptation Model as a conceptual framework to guide their intervention of a seated exercise program [41]. One further study, investigating a mindful yoga-based intervention, described their rationale as increasing psychological processes (e.g. acceptance, mindfulness) to improve cancer-related pain, fatigue, and distress [40].

**Table 3:**
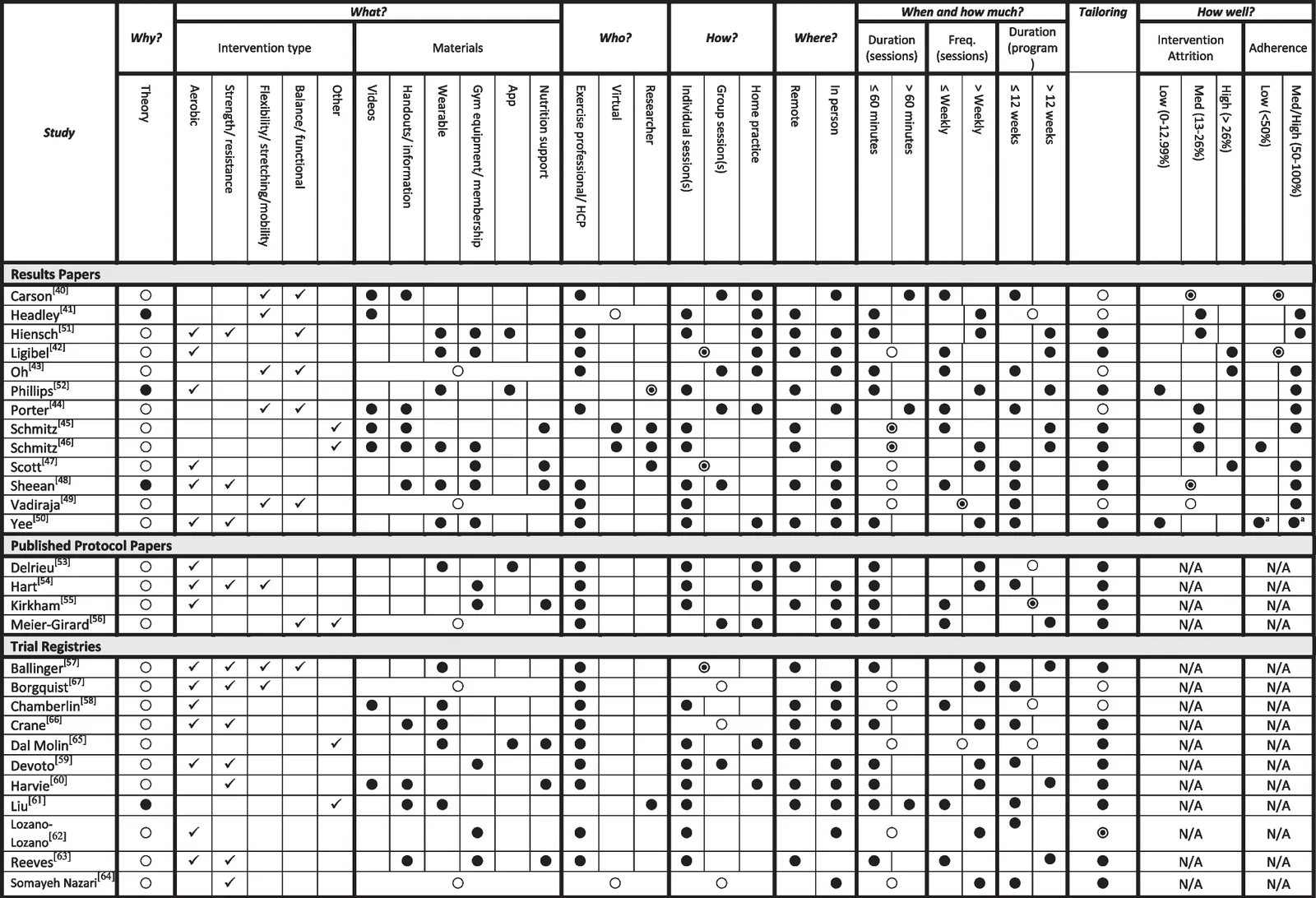
Summary of interventions according to the TIDieR checklist

= reported.  = not reported.  = unclear. aYee et al., (2019). Adherence to the resistance component was 100%; adherence to the walking component was 25%

Six interventions involved one type of PA [41, 42, 45–47, 52] and six involved two [40, 43, 44, 48–50]. The most commonly studied type of PA was aerobic [42, 47, 48, 50–52], followed by flexibility, stretching and mobility [40, 41, 43, 44, 49], and balance/functional activity [40, 43, 44, 49, 51]. Only three full reports evaluated an intervention involving resistance/strength exercises [48, 50, 51], and two reports of the same intervention evaluated a virtual assistant [45, 46]. Most interventions used a combination of materials; five used videos [40, 41, 44–46], five used handouts/information materials [40, 44–46, 48], and six provided some form of gym equipment or membership [42, 46–48, 50, 51]. Six interventions used a wearable device [42, 46, 48, 50–52], and three provided nutrition support in addition to PA [45, 47, 48].

Eight interventions were delivered by an exercise or healthcare professional, including yoga instructors [40, 44, 49], trained exercise specialists/physiologists [42, 48, 50, 51], and an experienced medical Qigong instructor [43]. Four interventions were delivered by a member of the research team [45–47, 52], and two used virtual assistants [45, 46]. Eight interventions were delivered individually [41, 45, 46, 48–52], three via group sessions [40, 43, 44], and one as a mixture of individual sessions and group cooking classes [48]. Seven interventions explicitly mentioned some element of home practice [40–44, 50, 51]. Four interventions were delivered exclusively remotely [41, 45, 46, 52], five in person [40, 43, 44, 47, 49], and four were a mixture of in person and remote [42, 48, 50, 51].

Four articles did not report the duration of sessions [42, 47–49], and two were unclear [45, 46]. Where duration was reported, two interventions involved sessions of > 60 min [40, 44], and five involved sessions ≤ 60 min [41, 43, 50–52]. Six interventions had sessions occurring weekly or less frequently [40, 42–45, 48], and seven lasted for ≤ 12 weeks [40, 43, 44, 47–50]. Tailoring was reported in eight interventions, often described as individualising the exercise based on performance, strength, progression, symptoms, and metastases location [42, 45–48, 50–52].

Medium to high (≥ 50%) adherence was reported in most studies (*n* = 10) [41, 43–45, 47–52], but it was unclear in two [40, 42]. In one study, adherence to 16 supervised sessions involving a brisk walk and resistance training was 100%, but only 25% of women adhered to the accompanying, unsupervised, walking component [50]. Attrition was varied, with two studies reporting low [50, 52], five medium (13 to 26%) [41, 44–46, 51], and three reporting high levels of attrition (> 26%) [42, 43, 47]. The highest attrition rate was 37%, from a study evaluating a Medical Qigong intervention [43].

### Study findings

#### Primary outcome of interest: physical activity

A wide variety of physical activity measures were used across trials. Increases in time spent exercising were reported in four studies [42, 48, 51, 52], with two being statistically significant [48, 51], despite none being powered to detect a significant effect on these outcomes. Significant improvements were reported for handgrip strength, physical fitness, and additional physical assessments (e.g. 6-minute walk test, leg strength) across three studies of varying sizes (*n*’s = 14, 40, 357) [48, 50, 51]. All three studies included both aerobic and resistance training exercises, with some supervision.

#### Secondary outcomes of interest

Five studies reported on sleep outcomes [44–47, 52], with none reporting significant between-group differences, despite some trends towards improvement [52]. All were underpowered to detect effects on these outcomes.

Evidence for interventions improving fatigue was mixed: four studies, testing yoga [49], seated exercise [41], supervised resistance training plus unsupervised walking [50], and a large scale (*n* = 357) 9-month aerobic, resistance, and balance programme [51], found statistically significant improvements. A further three trials reported non-significant trends and/or improvements pre-post intervention, but not between groups [42, 48, 52]. The largest trial (*n* = 357) found significant improvements in fatigue at 3, 6, and 9 months post-randomisation (effect sizes = 0.14 to 0.24) [51]. This trial also reported significant improvements in health-related QoL and physical fitness at all follow-ups, alongside improvements in physical functioning at 6 and 9 months. Two studies, testing aerobic exercise [42], and an in-home mHealth intervention [46], did not improve fatigue.

Health-related quality of life, measured by the EORTC QLQ C30 or FACT-B, was significantly improved in two studies, involving both aerobic and resistance training [48, 51]. Non-significant trends were reported in a further three (involving aerobic only/and resistance training) on global health scores and various subscales [42, 50, 52]. However three studies, involving Qigong [43], cycling [47], and a virtual assistant [45], reported no significant differences on the FACT-B and/or SF36. One [47] reported the attention control group (stretching exercises) significantly increased scores in the FACT-general score compared with those in the aerobic exercise group.

The majority (*n* = 9) of studies reported no safety events and/or additional healthcare visits related to the intervention [41–46, 48, 50, 52], and two did not report on safety [40, 49]. One pilot study reported that 36% of participants in the aerobic exercise intervention arm stopped a session early due to non-serious health events [47], 73% (*n* = 24) experienced at least one non-serious adverse event (AE) (compared to 7 AEs occurring in the attention control (stretching) group), and zero serious adverse events (SAEs) were observed. One phase III trial testing resistance training, aerobic exercise, and balance reported two exercise-related SAEs (a wrist fracture and sacral stress fracture), and 80 AEs requiring modifications to the exercise programme [51]. It was not specified whether those AEs were related to the intervention.

Four studies included clinical outcome measures (e.g. inflammatory biomarkers); however, these were not all reported within the included articles [41, 43, 47, 48]. Of those that were, one reported no difference in tumour response to treatment between groups [41], another reported no differences in haematological profiles between groups [47], and one subgroup analysis (*n* = 12) reported a non-significant increase in respiratory capacity in intervention participants [48].

### Protocols and study registries

#### Characteristics of studies

We identified 15 ongoing trials. Most are being conducted in the USA (3) [57, 58, 66], Europe (5) [53, 56, 60, 62, 67], and Australia (4) [54, 59, 61, 63]. Studies are also based in Iran [64], Brazil [65], and Canada [55]. Four studies are phase III RCTs [53, 56, 63, 66], seven are phase II or pilot/feasibility studies [54, 55, 57, 58, 60, 61, 67], and the remainder did not describe the phase [59, 62, 64, 65]. The planned or actual sample sizes range from 4 [58] to 260 [66] participants. Six studies will include individuals with an ECOG performance status of ≤ 2 [53, 55, 57, 59, 61, 63], four studies with an ECOG of ≤ 1 [56, 60, 62, 67], and one with a Karnofsky performance status of ≥ 80% [58]. The primary outcomes will include self-reported measures of fatigue [53, 54, 56, 59, 66] and quality of life [53, 62, 65], and self-reported [58, 61, 63] and objective [57, 58, 61, 63] assessments of physical functioning/activity. Six studies are using co-primary outcomes: one combining self-reported fatigue with either time to deterioration of health status or HrQoL [53], one assessing self-reported physical function and whole body lean mass [63], one assessing objective functional capacity alongside self-reported QoL [62], and two using objective and self-reported assessments of physical functioning/activity [58, 61]. Other primary outcomes include progression-free survival [60], liver enzymes [64], change in lesion size [55], feasibility/acceptability [54, 67], and safety [54]. Follow-up periods range from 8 weeks [64] to 2 years [55].

Three studies are using usual care as a comparator [54, 58, 62]; six are using active controls [56, 59–61, 65, 66]. Four [53, 55, 57, 63] are using varying approaches to enhanced usual care, e.g. a leaflet [57] or PA recommendation [53].

#### Intervention characteristics according to the TIDieR checklist

Table 3 summarises the intervention characteristics for trial registries and protocols, according to the TIDieR checklist, with full data extraction notes in Supplementary File 3. One report states that topics of discussion in behavioural counselling will be guided by the transtheoretical model of behaviour change [61]. No others describe a theoretical basis for intervention development. Eight interventions are incorporating one type of PA [53, 55, 58, 60–62, 64, 65], and four are incorporating two types [56, 59, 63, 66]. Aerobic exercise is the most common (*n* = 10) [53–55, 57–59, 62, 63, 66, 67], followed by strength/resistance training (*n* = 8) [54, 57, 59, 60, 63, 64, 66, 67]. Six interventions are incorporating wearable devices [53, 57, 58, 61, 65, 66], five include access to gym equipment or a membership [54, 55, 59, 62, 63], and two involve an app [53, 65].

Most interventions (*n* = 13) are being delivered by exercise professionals or healthcare professionals including physiotherapists/exercise professionals [53–55, 57, 59, 60, 63, 65, 67], dieticians/nutritionists [55, 60, 63, 65], nurses [58, 65], psychologists [65], a multidisciplinary team [62], a trained health coach [66], and a certified eurythmy therapist [56]. One intervention is being delivered by a doctoral student [61].

Nine interventions are individual [53–55, 58, 60–63, 65], one involves small groups of 1–4 people [56], and one includes either individual or group sessions of up to four people [59]. Five interventions are encouraging home practice [53, 54, 56, 60, 65]. Four interventions are remote [53, 57, 63, 65], six are in person [54, 56, 59, 62, 64, 67], two are using a combination of in person and remote sessions [55, 61], and three are offering a choice [58, 60, 66].

Most interventions include sessions > 60 min (*n* = 10) [53–57, 59–61, 63, 66], with one including longer sessions (90–120 min) for the first and final session [61]. Five interventions have sessions occurring weekly or less frequently [55, 56, 58, 61, 63], while sessions are held more than weekly for nine interventions [53–55, 59, 60, 62, 64, 66, 67], taking place up to three times a week [54, 57, 64, 66, 67]. The duration of interventions ranges from 6 weeks [59] to 12 months [63].

Most interventions report tailoring; five describe tailoring based on a combination of factors including metabolic equivalent time minutes, treatment regime, current activity and energy levels, heart rate, and performance [53, 60, 63]. One focuses tailoring on heart rate, rate of perceived exertion, and responses during the session [57], five describe tailoring based on individual capabilities and needs [56, 59, 61, 64, 66], one describes providing personalised advice in a chat [65], and one states the intervention is individualised but does not specify how [62]. Two interventions do not report any tailoring [58, 67].

## Discussion

This systematic review demonstrates that PA interventions in people with advanced and MBC are safe, well adhered to, and have the potential to improve fatigue, health-related QoL, physical fitness, and physical functioning over the short and medium term. However, as many trials are still ongoing and most completed studies only tested for feasibility, evidence-based recommendations on frequency, duration, delivery mode, and intensity cannot yet be made. Furthermore, trials with longer follow-ups (> 9 months) are needed to understand if activity levels and improved outcomes are sustained after intervention periods are completed. The overall methodological quality of the completed studies was moderate. While most employed appropriate study designs, outcome measures, and randomisation procedures, common limitations included differential treatment between groups, inadequate reporting on allocation concealment of outcome assessors, and unclear reporting of group differences on follow-ups.

The type of interventions being evaluated varies from yoga, walking, and strength training, to in-home mHealth personal assistants. Interventions that included both a strength/resistance element and an aerobic element seemed to be more effective at improving fatigue, QoL, and fitness than other activity types. Adherence levels were high overall, but dropped off over time and were generally lower for unsupervised components (mHealth [45] and walking [50]). The variety in activity type reflects the current lack of clinical guidance. Furthermore, a ‘one-size-fits-all’ approach is unlikely to be effective in this group, given the vast individual differences in physical capabilities, symptoms, and preferences [30, 68]. Literature from other populations emphasises enjoyment as key to adherence [69, 70], so patient preferences and individual barriers and facilitators to being active should be considered. While individual tailoring was evident in most studies, the specifics were not always reported. Information on the development of existing interventions is sparse, with only four stating some theoretical basis. Intervention development guidance advocates for theory-based interventions, to increase our understanding of the mechanisms of action [71] and increase effectiveness: PA interventions using techniques linked to Control Theory [72] are twice as effective as other interventions [73]. Future interventions should have a clearly described and reported theoretical basis.

The field of PA in MBC is growing, with 15 ongoing trials identified over the last 13 years. Within these ongoing trials there has been a greater emphasis on evaluating interventions which include strength/resistance training. This reflects the growing recognition of the importance of strength training for individuals with cancer, including breast cancer, to reduce the risk of cancer-related fatigue, muscle loss, and impaired QoL [14]. Early evidence from a small number of completed studies in this review suggests interventions involving strength training components could also improve fatigue and QoL in MBC [48, 50, 51].

### Strengths

This is the first systematic review looking at PA specifically in MBCs. All screening, data extraction, and quality assessments were conducted in duplicate. Including protocols and ongoing trials ensured a comprehensive overview of the current research in this field, providing a single resource for researchers, clinicians, and affected individuals.

### Limitations

Non-English language publications were excluded. We did not extract information on behaviour change techniques (BCTs) [74, 75] as this was beyond the scope of this review, and most papers did not explicitly report them. Therefore, the granular level of intervention content from a behaviour change perspective is missing. Broader PA interventions, such as lifestyle-integrated activities (e.g. gardening), may have been missed, as these were not explicitly listed within our search terms.

### Implications for clinical practice

Evidence to date suggests women with MBC should be encouraged to be active, and offered access to appropriate programmes that include both aerobic and strength/resistance-based exercises. Consideration of how to implement effective interventions within healthcare services is needed. Existing studies suggest that tailored interventions delivered by qualified exercise professionals can improve various outcomes, and are acceptable to those participating. Delivery by qualified exercise professionals may be particularly important for programmes incorporating a strength training element, to ensure individual modifications can be made for safety. Evidence-based recommendations on specific frequency, duration, delivery mode, and intensity cannot yet be made. The accessibility of available PA programmes should be considered when making recommendations, to ensure potential inequalities are not increased, for example highlighting online programmes for those in rural areas or arranging for translators to overcome language barriers.

### Implications for future research

Fully powered definitive trials, with cost-effectiveness evaluations, longer term (> 9 months) follow-ups, and that properly evaluate all intervention components, are required. Interventions designed with patient and healthcare professionals’ involvement, which have a theoretical basis, are also needed. Most interventions were tested in parallel group designs or with crossover arms, which cannot provide evidence on whether all, or only some intervention components are effective. Future trials could consider novel trial designs (e.g. complex factorial trials) within the MOST framework to more efficiently design, optimise, and evaluate multi-component interventions [76]. Consistency of outcome measures across trials would increase our understanding of the effectiveness of PA on different outcomes. Future studies could include descriptions of the BCTs used, to enhance our knowledge of the active components of interventions [77]. Improved reporting of participant characteristics is needed to assess sample representativeness and whether certain groups are being excluded. Those that did report participant characteristics included samples that were not very diverse, with most participants being 50–60 years old and White.

## Conclusion

Evidence on the efficacy and safety of physical activity for women with MBC is growing. More trials are required before specific, evidence-based recommendations on PA type, duration, mode of delivery, and frequency can be made, along with their long-term effectiveness on clinical outcomes. However, existing studies provide initial evidence that exercise can be safe, well adhered to, and improve various outcomes including fatigue and QoL, for women with MBC. Physical activity should be actively encouraged and adequately supported by healthcare professionals involved in their care.

## Supplementary Information

Below is the link to the electronic supplementary material.

ESM1 (DOCX 91.0 KB)

## Data Availability

Eligibility criteria and full search strategies for all databases can be found on the Open Science Framework:https://doi.org/10.17605/OSF.IO/2SW76.
