# Supplementary material for: Physical activity interventions for women with metastatic breast cancer: a systematic review of published and ongoing randomised controlled trials
Source: J Cancer Surviv. 2025 Nov 11;20(5):2710–29. doi: 10.1007/s11764-025-01917-y (PMC13723549; doi:10.1007/s11764-025-01917-y)
Supplement: Supplementary file 1 [file 11764_2025_1917_MOESM1_ESM.docx]

**Supplementary Files**

**Physical activity interventions for women with metastatic breast cancer: A systematic review of published and ongoing randomised controlled trials**

Louise H. Hall, Sophie M.C. Green, Zainab Haider, Abigail Fisher, Natalie King, Rebecca J Beeken^*^, Samuel G. Smith^*^

*=Contributed equally to the work and should be considered joint senior authors

**Journal of Cancer Survivorship

Corresponding author**
Professor Samuel G. Smith, University of Leeds

Email: s.smith1@leeds.ac.uk

**Supplementary File 1. PRISMA 2020 Checklist**

| **Section and Topic** | **Item #** | **Checklist item** | **Location where item is reported** |
| --- | --- | --- | --- |
| **TITLE** | | |  |
| Title | 1 | Identify the report as a systematic review. | 1 |
| **ABSTRACT** | | |  |
| Abstract | 2 | See the PRISMA 2020 for Abstracts checklist. | 2 |
| **INTRODUCTION** | | |  |
| Rationale | 3 | Describe the rationale for the review in the context of existing knowledge. | 3, 4 |
| Objectives | 4 | Provide an explicit statement of the objective(s) or question(s) the review addresses. | 4 |
| **METHODS** | | |  |
| Eligibility criteria | 5 | Specify the inclusion and exclusion criteria for the review and how studies were grouped for the syntheses. | 5, 6 |
| Information sources | 6 | Specify all databases, registers, websites, organisations, reference lists and other sources searched or consulted to identify studies. Specify the date when each source was last searched or consulted. | 4, 5 |
| Search strategy | 7 | Present the full search strategies for all databases, registers and websites, including any filters and limits used. | Appendix 2, OSF |
| Selection process | 8 | Specify the methods used to decide whether a study met the inclusion criteria of the review, including how many reviewers screened each record and each report retrieved, whether they worked independently, and if applicable, details of automation tools used in the process. | 5 |
| Data collection process | 9 | Specify the methods used to collect data from reports, including how many reviewers collected data from each report, whether they worked independently, any processes for obtaining or confirming data from study investigators, and if applicable, details of automation tools used in the process. | 5, 6 |
| Data items | 10a | List and define all outcomes for which data were sought. Specify whether all results that were compatible with each outcome domain in each study were sought (e.g. for all measures, time points, analyses), and if not, the methods used to decide which results to collect. | 6 |
|  | 10b | List and define all other variables for which data were sought (e.g. participant and intervention characteristics, funding sources). Describe any assumptions made about any missing or unclear information. | 6 |
| Study risk of bias assessment | 11 | Specify the methods used to assess risk of bias in the included studies, including details of the tool(s) used, how many reviewers assessed each study and whether they worked independently, and if applicable, details of automation tools used in the process. | 6 |
| Effect measures | 12 | Specify for each outcome the effect measure(s) (e.g. risk ratio, mean difference) used in the synthesis or presentation of results. | Table 1 |
| Synthesis methods | 13a | Describe the processes used to decide which studies were eligible for each synthesis (e.g. tabulating the study intervention characteristics and comparing against the planned groups for each synthesis (item #5)). | 6 |
|  | 13b | Describe any methods required to prepare the data for presentation or synthesis, such as handling of missing summary statistics, or data conversions. | n/a |
|  | 13c | Describe any methods used to tabulate or visually display results of individual studies and syntheses. | 6, 7 |
|  | 13d | Describe any methods used to synthesize results and provide a rationale for the choice(s). If meta-analysis was performed, describe the model(s), method(s) to identify the presence and extent of statistical heterogeneity, and software package(s) used. | 6, 7 |
|  | 13e | Describe any methods used to explore possible causes of heterogeneity among study results (e.g. subgroup analysis, meta-regression). | n/a |
|  | 13f | Describe any sensitivity analyses conducted to assess robustness of the synthesized results. | n/a |
| Reporting bias assessment | 14 | Describe any methods used to assess risk of bias due to missing results in a synthesis (arising from reporting biases). | n/a |
| Certainty assessment | 15 | Describe any methods used to assess certainty (or confidence) in the body of evidence for an outcome. | n/a |
| **RESULTS** | | |  |
| Study selection | 16a | Describe the results of the search and selection process, from the number of records identified in the search to the number of studies included in the review, ideally using a flow diagram. | 7, Figure 1 |
|  | 16b | Cite studies that might appear to meet the inclusion criteria, but which were excluded, and explain why they were excluded. | n/a |
| Study characteristics | 17 | Cite each included study and present its characteristics. | 8, 9, 13, Table 1, Table 3 |
| Risk of bias in studies | 18 | Present assessments of risk of bias for each included study. | 7, 8, Table 2 |
| Results of individual studies | 19 | For all outcomes, present, for each study: (a) summary statistics for each group (where appropriate) and (b) an effect estimate and its precision (e.g. confidence/credible interval), ideally using structured tables or plots. | 11, 12, Table 1 |
| Results of syntheses | 20a | For each synthesis, briefly summarise the characteristics and risk of bias among contributing studies. | 11, 12 |
|  | 20b | Present results of all statistical syntheses conducted. If meta-analysis was done, present for each the summary estimate and its precision (e.g. confidence/credible interval) and measures of statistical heterogeneity. If comparing groups, describe the direction of the effect. | n/a |
|  | 20c | Present results of all investigations of possible causes of heterogeneity among study results. | n/a |
|  | 20d | Present results of all sensitivity analyses conducted to assess the robustness of the synthesized results. | n/a |
| Reporting biases | 21 | Present assessments of risk of bias due to missing results (arising from reporting biases) for each synthesis assessed. | n/a |
| Certainty of evidence | 22 | Present assessments of certainty (or confidence) in the body of evidence for each outcome assessed. | n/a |
| **DISCUSSION** | | |  |
| Discussion | 23a | Provide a general interpretation of the results in the context of other evidence. | 15, 16 |
|  | 23b | Discuss any limitations of the evidence included in the review. | 15, 17 |
|  | 23c | Discuss any limitations of the review processes used. | 16, 17 |
|  | 23d | Discuss implications of the results for practice, policy, and future research. | 17, 18 |
| **OTHER INFORMATION** | | |  |
| Registration and protocol | 24a | Provide registration information for the review, including register name and registration number, or state that the review was not registered. | 4 |
|  | 24b | Indicate where the review protocol can be accessed, or state that a protocol was not prepared. | 4 |
|  | 24c | Describe and explain any amendments to information provided at registration or in the protocol. | n/a |
| Support | 25 | Describe sources of financial or non-financial support for the review, and the role of the funders or sponsors in the review. | 19 |
| Competing interests | 26 | Declare any competing interests of review authors. | 19 |
| Availability of data, code and other materials | 27 | Report which of the following are publicly available and where they can be found: template data collection forms; data extracted from included studies; data used for all analyses; analytic code; any other materials used in the review. | Not reported |

*From:*  Page MJ, McKenzie JE, Bossuyt PM, Boutron I, Hoffmann TC, Mulrow CD, et al. The PRISMA 2020 statement: an updated guideline for reporting systematic reviews. BMJ 2021;372:n71. doi: 10.1136/bmj.n71. This work is licensed under CC BY 4.0. To view a copy of this license, visit <https://creativecommons.org/licenses/by/4.0/>

**Supplementary File 2: Search Strategy for Medline**

1 exp Breast Neoplasms/ and exp Neoplasm Metastasis/

2 (metasta* adj4 (breast or mammary)).tw,kf.

3 ((advanced or "stage 4" or "stage IV") adj2 (breast or mammary)).tw,kf.

4: 1 or 2 or 3

5: exp Exercise/

6 exp Sports/

7 exp Dancing/

8 (physical adj3 activit*).tw,kf.

9 (exercise or exercises or exercising).tw,kf.

10 (physical adj3 (therap* or train* or conditioning)).tw,kf.

11 ((muscle or strength* or resistance or circuit* or interval or weight*) adj3 train*).tw,kf.

12 exp Swimming/

13 exp Walking/

67787

14 exp Running/

15 exp Jogging/

16 sport*.tw,kf.

17 exp Fitness Centers/

18 gym*.tw,kf.

19 aerobics.tw,kf.

20: 5 or 6 or 7 or 8 or 9 or 10 or 11 or 12 or 13 or 14 or 15 or 16 or 17 or 18

21 controlled clinical trial.pt.

22 randomized controlled trial.pt.

23 randomized.ab.

24 placebo.ab.

25 clinical trials as topic.sh.

26 randomly.ab.

27 trial.ti.

28: 21 or 22 or 23 or 24 or 25 or 26 or 27

29 exp animals/ not humans.sh.

30: 28 not 29

31: 4 and 20

32: 30 and 31

**Supplementary File 3: Data extraction for interventions according to the TIDieR checklist**

|  | **Name** | **Why** | **What (materials)** | **What (procedures)** | **Who provided** | **How** | **Where** | **When/how much** | **Tailoring** | **Modifications** | **How well (planned)** | **How well (actual): adherence and attrition** |
| --- | --- | --- | --- | --- | --- | --- | --- | --- | --- | --- | --- | --- |
| **Full papers** | | | | | | | | | | | | |
| Carson, 2021^[39]^ | Mindful Yoga | To address cancer-related pain, fatigue, and emotional distress. Rationales:  1) Cultivating an acceptance of and willingness to learn from pain and other stressful experiences, to decrease emotional distress and sympathetic activation associated with pain.  2) Yoga produces relaxation responses, which may promote alleviation of symptoms such as pain and fatigue.  3) Yoga postures serve as a forum for developing nonreactive awareness of bodily sensations, including pain and fatigue. Mindfulness strategies and acceptance to become aware of and modulate subtle patterns of unskilful reactivity (e.g. fear, guarding). | Professional video recordings guiding on meditation, breathing, and posture strategies.  Participants also received brief session summary handouts each week that included instructions for the informal application of yoga practice to daily life (e.g., in-the-moment acceptance of pain). | "Mindful Yoga program emphasizes developing mindfulness via substantial meditation (25 minutes per class), gentle postures (40 minutes), breathing techniques (10 minutes), presentations on yogic principles for optimal coping (20 minutes), and group discussions with opportunities for sharing and asking questions (25 minutes).”  Intervention instructors received prior training in the respective protocols and followed detailed treatment manuals. All sessions were video recorded and reviewed by investigators who provided verbal and written feedback to the instructors on a weekly basis. | Led by registered yoga teachers (i.e., registered yoga teacher [RYT] certification from Yoga Alliance) with prior experience in teaching yoga techniques to medical patients. | In-person groups | Not reported. Some home practice. | Each intervention consisted of eight 120-minute group sessions, with 5-10 patients per group. Sessions were generally held weekly. Recommendation for 15-30 minute home practice per day, 5-6 days per week | Not described. | Not described. | Adherence (amount of yoga practice) at home was assessed by daily diaries asking time spent on each of the three practices. | On average, participants spent 41 minutes doing a combination of meditation, breathing, and posture practices on the days diaries were completed. |
| Headley, 2004^[40]^ | Armchair Fitness: Gentle Exercise | Based on Roy's Adaption Model. Hypothesised the seated programme would alter participants' coping mechanisms which would result in decreased fatigue and improved quality of life. | Chair, video tape of exercise instructions/demonstrations, video player, TV. | Five minute warm up, 20 minutes of moderate intensity repetition motion exercises, 5-minute cool down.  Participants sit in a straight-backed chair while performing stretching and repeated flexion and extension of the arms, head, upper torso, and legs. The video has no activities for strength or resistance training; no equipment is used. | Unsupervised. Pre-existing commercially available video - not reported who the person recorded is | Individual, unsupervised, Remote (videos, not live). | Not reported | 30 minutes, 3 times a week (With at least a one-day break between sessions). | Not described | Not described | Subjects in both groups were asked to document on monthly calendar logs the dates, times, intensity, types, and lengths of any activities, including seated exercise. | Overall adherence rate was 75%, and declined over the 3-month period.  Attrition was 16% (n=6). |
| Hiensch, 2024^[50]^ | PREFERABLE-EFFECT | Exercise improves physical performance and functioning in patients with advanced cancer, and may improve other cancer and treatment related side-effects (but more evidence is required) | Physical activity tracker, resistance bands, free weights, cycle ergometer, aerobic step, rowing machine, treadmill, cross-trainer, app. | If live-online, trainer conducts one individual face to face exercise session to explain everything. During each session the trainer closely monitors each participant for safety. Balance training = 5 minutes of various exercises. Aerobic training = moderate intensity continuous training (MICT) and progresses to HIIT with at least one session per week on a cycle ergometer, the other session on the rowing machine, treadmill, or cross-training. Intensity gradually increased during the programme. Resistance training = 6 exercises targeting major lower and upper body muscle groups, using machine based weights, free weights, or body weight. Unsupervised = 60min session supported by the app: this is to replace one of the supervised sessions in the final 3 months. The app contains: exercises learned during the supervised programme and can be performed at home. Illustrated with simple animations and instructions. | Exercise professional (e.g. exercise physiologist or oncology physiotherapist) trained by the study team. | In person, supervised and unsupervised. Remote sessions available. | Community or hospital-based fitness centres, physical therapy practices, gyms, or personal training facilities. And/or at home via live-online training (e.g. due to COVID). | Supervised 2 x per week 60 minutes. Unsupervised encouraged to be active 30min daily. Then switch to 1x60 minutes of supervised and 1 x 60 minutes unsupervised for last 3 months of programme. For aerobic training the load should be decreased by 10% if the Rate of Perceived Exertion (RPE; 6-20) is higher than 15, whereas the load can be increased by 10% if the RPE is lower than 13. Exertion assessed by Borg scale. | Adapted for physical limitations. Aerobic training individualised based on their fitness level using the MSEC. Resistance exercise individualised based on fitness assessed by the training during the intake session using a 12 rep max. Those with bone mets perform aerobic training on the cycle ergometer or treadmill and do not conduct the 12RM test for exercises that load regions with bone mets. | Modified for those with AEs/SAEs but not described how. | Trainer documents attendance and compliance for each scheduled session. | The median (interquartile range (IQR)) attendance rate for supervised exercise sessions was 77% (48–92%). Median compliance with the exercise protocol was 70% for the balance component, 59%–83% for the aerobic components and 63%–100% for the resistance components.  The overall dropout rates were 12.3% at 3 months, 18.5% at 6 months and 24.1% at 9 months. |
| Ligibel, 2016^[41]^ | Metastatic Exercise Training Trial | Evidence for improved physical function and reduced fatigue in early-stage breast cancer, but little information on the feasibility and benefits of exercise in women with advanced breast cancer. | Heart rate monitor, pedometer, exercise journal, 16-week membership to a gym in their local area. | 16-week aerobic exercise program. Sessions focused on building exercise self-efficacy, overcoming barriers to exercise, documenting any injuries, and reviewing safe exercise practices. The target goal was 150 minutes of moderate intensity exercise per week. | Exercise physiologist. | In-person and telephone, educational sessions with unsupervised activity in-between. Not reported if individual or group-based. | Not reported | Weekly sessions for first month and monthly thereafter supplemented by weekly calls for 16 weeks total. Moderate intensity. | Exercise physiologist individualised the programme for each participant | Not described | Compliance was assessed through evaluation of weekly exercise journals and through monitoring attendance at supervised exercise sessions and telephone calls. | Average number of minutes of self-reported exercise per week in journals = 134.8 (± 138.3). Approximately 70% of the  women randomized to the exercise intervention completed the 16-week program. |
| Oh, 2014^[42]^ | Medical Qigong (MQ) | MQ improves QoL, mood, reduced fatigue, and depressive symptoms, improve cognitive function and reduce inflammatory biomarkers in cancer patients. Hopefully will be able to in MBC. | Not described | 10-minute discussion of health issues, 30mins gentle stretching and body movement in standing postures to stimulate energy channels; and 20 minutes meditation and breathing exercises based on the energy channel theory in Chinese medicine, including natural breathing, chest breathing, abdominal breathing, breathing for energy regulation, and relaxation and visualization. | Experienced MQ instructor with over 20 years’ experience and training in traditional Qigong in Korea, Daoist Qigong in China, Buddhist Qigong in Australia, and mind-body medicine at the Harvard Medical School. | Group supervised in-person class. | In their treating hospital. | 10 weeks with one supervised 60-minute session per week.  Participants were also encouraged to undertake home practice every day for at least half an hour. | Not described | Not described | Participants were considered to have completed the MQ and meditation program if they attended a minimum of five out of ten weeks. To assess home practice,  participants were asked how many days they practiced at home each week and duration of practice. | 80% adherence rate. Participants reported practicing at home 3-4 days/week for 15-30mins a day. Of the 27 women participating, 63% of participants completed the full 10 weeks of the study (MQ intervention (n=9) and meditation control (n=8)). |
| Phillips, 2024^[51]^ | Fit2ThriveMB | Increased PA associated with fewer side-effects, higher QoL, increased survival, reduced recurrence and mortality. mHealth to allow remote monitoring and tailoring. Conceptual model = Social Cognitive Theory; intervention aims to increase self-regulatory and goal-setting skills, increase self-efficacy via increased mastery experiences and social persuasion, increase realistic outcome expectations, improve facilitators by increasing social support, and increase the ability to overcome barriers, resulting in increased PA. Increased PA is, in turn, hypothesized to result in favourable changes in symptom burden, QoL, and functional performance. | Fit2ThriveMB app, FitBit. | Access to app for 12 weeks (and further 9 months), plus coaching calls. Participants will be encouraged to accumulate more steps throughout the day by (1) moving more and sitting less, (2) adding planned structured exercise sessions to their day, or (3) a combination of (1) & (2). They will also be provided with a more detailed exercise prescription if they decide to adopt approach (2). The App encourages increasing PA through SCT principles including goal setting, self-monitoring, personalised feedback on progress, app notifications, education on BCTs, social support. App includes social feed where they can communicate with 5-8 other participants, moderated by a study team members. During weeks 1-7 of the intervention, there will be 2 pre-planned posts in the social feed. During weeks 8-12, the study staff will post 1 pre-planned post a week in the social feed. Semi-structured weekly coaching calls provide feedback on symptom burden and progress on goals, review goals, strategies for increasing PA, and covers at least 1 SCT behaviour change strategy. Also told to call or email their coaches throughout the week if they have any issues. | Study team -Not described (Doesn't report profession / experience of persons delivering coaching calls.) | App and phone calls | Remotely delivered app and phone calls: exercise taking place wherever participants choose. | Weekly coaching calls lasting 10-15mins. Supported app for 12 weeks (but access for 12 months). | Tailored goal setting and exercise prescription based on maximum heart rate. | N/A | Fitbit usage: average number of days of wearing the Fitbit (≥2000 steps/day) and proportion of days of wearing the Fitbit.  Fit2ThriveMB app: average number of days participants opened the app and average time spent using the app each day.  Daily goal setting: average number of days participants responded to the survey  Fit2ThriveMB app social feed: total number of social feed posts; the average number of days of access to the social feed page.  • Fit2ThriveMB app notifications: average number of messages read.  • Fit2ThriveMB coaching calls: total percentage of coaching calls attended; average time per call; fidelity to coaching call script. | Step goal met on 53.4% of study days. App opened on 94.1% of study days. Symptom burden rating and goal self-selection on 87.4% of study days. Fitbit worn on 92.7% of study days. 98.3% of coaching calls attended. Average call length was 22 min (SD 15.0). 30.4% of app notifications marked as read. Posted on social feed average of 16.2 times.  Attrition: 1 (2%) (deceased). |
| Porter, 2019^[43]^ | Mindful Yoga | Yoga may improve psychosocial outcomes and functional wellbeing | Yoga videos and handouts of instructions for applying yoga practice in everyday life. | Sessions = 40mins gentle postures, 10mins breathing techniques, 25mins meditation, 20mins presentations on the application of yogic principles to optimal coping, 25min group discussions. Encouraged to practice at home. | Certified yoga instructor | In person. Group sessions of 5-8 patients per group, plus home practice. | Not reported. Some home practice. | 8 x 120 min weekly group session. | Not described | Not described | All sessions video recorded and reviewed by investigators who provided feedback to the instructors weekly. Participant adherence to intervention monitored. | 65% attended >=4 sessions. Mean attendance was 4.6 (SD=2.9) sessions. 6 participants in yoga condition withdrew, 1 died, and 3 lost to contact (out of 43). |
| Schmitz, 2023^[44]^ | Nurse AMIE (for Alexa) | Tech-based interventions can promote health behaviours and support women with MBCs unmet needs, to improve symptom burden and QoL. Adaptation of previous study using same concept but, on a tablet, - change to a conversational user interface to reduce intimidation of using a tablet. | Amazon Echo Show with Alexa, with the programme Nurse AMIE programmed into it. | Access to ALEXA and Nurse AMIE for 6 months. Nurse AMIE was programmed to give a greeting each day with a nutrition tip and recipe (based on information found on the American Institute for Cancer Research Website). Then it asks pain, sleep, fatigue, distress, and step counts from yesterday. It then gives an empathic response. If pain or distress is 7+/10, they are reminded to contact their clinical team (and a notification sent to the study team, who then contacted the oncology care team). Based on symptom rating, nurse AMIE recommended an activity to help with symptoms, which could include: soothing music, CBT lessons, guided relaxation, exercise videos (balance, strengthening, stretching), or audio messages to assist with symptom management. Participants could choose what they wanted from an activity menu. Weekly calls from study team for first 3 months. | Virtual assistant (Alexa) | Remote, individual | At home | Access for 6 months. Weekly calls, for 3 months from the study team to enquire on wellbeing, symptom checks, troubleshooting technology issues. No calls in months 4-6. | Intervention recommendations tailored using in-built algorithm designed by study team. Daily tailoring, based on person's reported symptoms and severity | Not described | Number of days interacting with it. Logins monitored. | Participants who participated in 90 days of the intervention (n=37), 24 (64.9%) logged in at least 30 days. Use of the technology declined in months 4–6, after the weekly calls ended: 58%, 47%, and 29% logged in during months 4, 5, and 6. 2 participants lost to follow up and one discontinued study in the immediate condition. |
| Schmitz, 2021^[45]^ | Nurse AMIE (for tablets) | Supportive tech-based interventions not requiring on-site appointment could be helpful for symptom management. | Tablet, pedometer, exercise band, binder with instructions. | Daily Nurse AMIE asks symptom questions, then responds accordingly. Data from pedometer and daily symptom questions for the past 3 days are applied to an algorithm that then gives tailored evidence-based interventions to address symptoms. Interventions include: CBT, guided imagery, mindfulness meditation, music, PA, guided relaxation, energy conservation, psychoeducation, social support (advice taken from Clinical Guidelines). Weekly patient navigator calls review symptoms, steps, and intervention satisfaction, discern whether new or different interventions are needed, and connect women to services if additional care is warranted. | Virtual assistant (Alexa) and patient navigator | Individual, eHealth (remote), plus telephone calls | Medical Centre | Daily for 6 months. Weekly check-ins for months 1-3. | Exercise programme customized according to presence and location of bone mets. Daily tailoring of intervention based on symptoms reported, and pedometer data. | Not described | Use of tablet (days used out of 30) | Average number of days patients logged in was 40.3 out of 90 Of the 17 patients who actively participated in using the intervention, 13 (76%) were still using it after 1 month. |
| Scott, 2018^[46]^ | Aerobic training | Mitigate treatment and disease related symptoms, and potentially improve disease outcomes. | Treadmill, stationary cycle ergometer. | "The intensity of each session alternated between 4 different dose intensities (i.e., 55%, 65%, 75%, and 80%) of the maximal metabolic equivalent (MET) (i.e., the peak oxygen consumption [VO2peak]". "Exercise dose (a function of training intensity and duration) was scheduled such that physiological stress was continually altered and progressively increased in conjunction with appropriate rest and recovery sessions to optimize physiological adaptation across the entire intervention period (i.e., non-linear periodized prescription). The dose and scheduling of sessions was standardized across all patients." | Study personnel with bachelor’s degree in exercise science. | Supervised, in person. Not reported if individual or group. | Exercise sessions = clinic-based. Cooking classes = community location. | 36 sessions thrice weekly for 12 consecutive weeks. | "The intensity was tailored to each patient on the basis of the workload (i.e., treadmill speed/grade) corresponding to a specific percentage of ventilatory thresholds measured during the pre-randomization or midpoint (week 6) CPET." Dose modification of any session was permitted and performed by the exercise physiologist monitoring each session according to standardized criteria". Some individual tailoring e.g. two patients move to from walking treadmill to cycle ergometer | Not described | Adherence to the protocol evaluated via standardized recording of session attendance and adherence to the planned dose/session. | 1 (3%) LTF. Mean session attendance = 63 ± 30. 9 (27%) permanent discontinuation. 15 (46%) dose interruptions. 16 (49%) dose modification. 6 (18%) pre-treatment dose modification. 12 (36%) early session termination. Mean Relative Dose Intensity of 61% ± 30%. |
| Sheean, 2021^[47]^ | Every Day Counts | Social cognitive theory used, but not justified. | Programme binder / materials addresses nutritional knowledge, attitudes and behavioural strategies, and recipes for plant-based eating, a physical activity self-monitoring form. Fitbit tracker, exercise bands, cooking utensils. 2 SMS per week on self-efficacy, outcome expectations, social support, and self-monitoring tools. | "Behavioural interventions addressing adoption of ACS nutrition and PA guidelines for cancer survivors. Aerobic and resistance exercise training sessions, individual telephone sessions based on the EDC program intervention manual, and intervention support. 1st supervised intervention session provided nutrition education and PA/RET guidance. The binder addressed knowledge, attitudes (barriers and facilitators), behavioural strategies, recipes, plant-based eating and PA self-monitoring form, and other materials to support adherence reinforcement and review.  Phone sessions began with info on weekly theme, discussion of previous week's achievements and challenges, review of adherence using the binder tracking form, and setting one SMART weekly goal for nutrition and one for PA/RET.  Supervised exercise sessions included: (1) guiding participant in her choice of appropriate modes of moderate PA to work toward engaging in 30 min most days of the week (e.g., walking, biking), (2) assessing proper type and intensity of RET for safe conditioning, and (3) instructing and observing participants in the correct execution of RET. Guidance also provided on endurance activities e.g. increasing step goals/time walking.  SMS messages: focused on self-efficacy, outcomes expectations, social support and/or resources or self-monitoring tools.  Optional cooking class: focused on simple plant-based  meals (1 salad, 1 side dish, 1 main course) in order to increase skills and comfort with preparing plant-based meals and promote dietary adherence" | Certified, licensed athletic trainer (LAT, ATC, CES) experienced in lifestyle counselling. | Supervised, in person, individual. Telephone sessions. Cooking classes were remote, and group-based. | Not described | 4 supervised exercise sessions over 12 weeks. Weekly telephone sessions. Weekly SMS messages. Opportunity to attend 3 cooking classes (group classes). | Resistance training tailored to participant's strength based on baseline physical assessment data and guided by normative score for strength. Progression of resistance exercises tailored based on participant feedback, consistency of program adherence via verbal feedback, and data from tracking device. | Not described. | Attendance for in-person & telephone sessions monitored. | Overall attendance was 86% during the 12-week intervention: 93% for in-person sessions and 84% for telephone sessions. Other components not reported (e.g. Cooking classes). |
| Vadiraja, 2017^[48]^ | Integrated yoga program | Yoga may be indicated for managing fatigue. | Not described | "An integrated yoga module comprising various practices that include asana (postures), pranayama (regulated nostril breathing), yogic relaxation in supine (shavasana), meditation, self‑appraisal, and counselling. Practices such as pranic energization technique (positron emission tomography ‑ observing the flow of energy or prana through the body), cyclic meditation ‑ combination of postures and relaxation techniques done keeping eyes closed, and mind sound resonance technique (chanting of mantras verbally and mentally) were some of the specific techniques used in cancer patients." Education was also used. | Trained yoga therapist | Individual supervised in-person sessions | Hospital | 24 sessions over a 3-month period | Not described | Not described | Session attendance | 80% of participants attended all 24 supervised sessions |
| Yee, 2019^[49]^ | Not described | PA may reduce symptom burden (e.g. fatigue, pain) and improve QoL. Delivered at home to reduce barriers to implementation, and due to preferences of the patient group. | Portable bar, resistance bands, free weights, pedometer. | "Supervised and unsupervised sessions. Supervised sessions involved brisk walk and resistance training including chest press, horizontal row, upright row, bicep curl, calf raises, lunges and sit-to stands or squats.  Women were asked to walk on days they were not seeing the trainer and encouraged to increase the steps taken each week by 10%". | University-trained exercise specialist | Individualised. Mixed in-person supervised and unsupervised | Participant's home or local park and in the community | 8-week programme of 16 exercise sessions, plus walking programme outside of those sessions (on days they don't have a supervised session (so 5/7)). Called by a researcher weekly. Supervised sessions lasted 10-15 minutes (brisk walk) and 30-40 minutes (resistance training). The short walk was monitored via a pedometer and Borg’s rating of perceived exertion, with a target of 11-13 to reflect a moderate intensity. Resistance exercises were 2 sets of 10-12 repetitions, with one-minute recovery between sets. Resistance training performed at a moderate intensity, targeting 6/7 out of 10 on the Adult OMNI Perceived Exertion Scale. | Each exercise was individualized based on training experience and baseline strength. Increase in steps each week based on individuals steps the previous week (increase by 10% each week). | Not described | Exercise diaries maintained by the trainer in each session. | All eight women attended 100% of the 16 supervised sessions.  All sessions completed at the prescribed intensity and volume. Only 2 women adhered to the walking program. Compliance with walking intensity was found in 71% of participants; No participants achieved the desired volume of walking. Retention was good in both exercise and control groups (100% and 83% respectively). No participants in intervention group were lost to follow up or discontinued intervention. |
| **Protocols** | | | | | | | | | | | | |
| Delrieu, 2020^[52]^ | ABLE02 | Activity trackers to motivate participants to be active, and to increase PA. PA to improve quality of sleep. PA to limit decline in muscle mass (sarcopenia) and therefore limit decline in HRQoL and survival. | ABLE02 mobile app (on smartphone or tablet) for questionnaire completion, and for intervention arm to monitor daily steps. Intervention arm only: Activity Tracker (Withings) to monitor steps and sleep, along with the Health Mate Withings App which included weekly quizzes on PA and nutrition. | One meeting with a PA instructor to receive international PA recommendations (both arms). Intervention arm also receive phone calls (monthly) to review and adapt step goals. Intervention arm also had a messaging system and phone line available. | Physical Activity Instructor | Individual, Remote (phone calls and app) | At home | Encouraged to have 3 walking sessions per week of at least 10 minutes. Phone call after 15 days post-randomisation and then monthly for goal setting. Encouraged to achieve daily step count goals. | Number of recommended steps per day is individualised and determined according to the 6MWD at baseline, and to participants’ preferences and capabilities. Data from 6MWD split into tertiles and recommendations adapted from Tudor Locke's paper. (Participants who achieve less than 399 m at the 6MWD will be encouraged to perform more than 2500 steps per day, between 400 and 455 m will correspond to a goal of 5000 steps per day, between 456 and 509 m will correspond to 7500 steps per day and performance above 510 m to a goal of 10,000 steps per day).  This will then be reviewed and adapted further during phone calls with the PA instructor at 15 days post randomisation, then monthly until the end of intervention (6 months). During these subsequent calls, the steps objective will be adapted based on number of steps performed in previous week and other parameters e.g. side-effects, patient preferences. | N/A | "The observance of the intervention will be evaluated by the number of patients who will have worn the activity tracker for the entire duration of the procedure and the number of patients who will have achieved their goal." The PA instructor will use a professional dashboard to access participants step counts and walking sessions from the ABLE02 app and monitor them from there. | N/A |
| Hart, 2018^[53]^ | M3EP-SIT | Exercise may up-regulate osteoblastic activity, allowing mechanically driven regulation of bone metabolism to counteract tumour-driven dysregulation. 1) preserve bone strength through anabolic morphological adaptations, 2) interfere with tumoral processes that blunt osteoblastic formation and promote osteoclastic resorption, thus supressing tumour growth in affected skeletal sties. | Treadmill, bike, rowing machine, exercise ball, exercise/resistance bands. | SIT followed by M3EP. SIT = spinal Isometric Training. M3EP = modular multi-modal exercise programme. M3EP = resistance, aerobic and flexibility exercises: participants perform 6 different resistance exercises using major muscle groups, at 10-12 reps for 3 sets per exercise (moderate intensity and volume). Aerobic exercises = treadmill, cycling, rowing, performed at 60-85% of HRMax for 20-30 minutes (using HR monitors). Flexibility = static stretching of muscles at all joints considered important for function, and for all muscles engaged during the sessions. Stretches are 2-4 sets per muscle group with 30-60second hold per set. SIT: isometric exercises designed to target and stimulate spinal lesion site(s) through muscle contraction - activate the full spinal column. 5 exercises in whole and partial weight-supported prone and supine positions on the floor with gentle dynamic accessory movements (can be seated or standing if required). Progression from beginner to advanced exercises will be individually determined. | Accredited exercise physiologist. | Individual, in person | In Clinic and at home. | 3X clinic sessions a week, for 12 weeks. Sessions last 60 minutes. Plus the SIT portion also performed at home 2x per week for 15 mins at a time. Participants will engage in cardiovascular exercise using various modes including treadmill, cycling and rowing ergometers, performed at 60–85% HRmax for 20–30 min using heart rate monitors. | Based on the location and extent of bone metastases. Aerobic exercises based on age-predicted HRmax, and tailored using heart rate monitors. SIT exercises can be on floor, seating, or standing and beginner to advanced depending on the individual. If floor exercises are contraindicated for the patient due to physical restrictions, alternate seated and standing isometric exercises will be provided. | N/A | Programme adherence and compliance assessed using an exercise diary completed by the patient at all clinic and home based sessions - data will be compared to their prescribed individualised exercise programme (completed versus missed sessions) and (prescribed versus actual exercise completed for each training mode) | N/A |
| Kirkham, 2021^[54]^ | DREAM | Fasting/change in diet prior to chemo can inhibit tumour growth, enhance chemo efficacy, and reduce side-effects, and increase the resistance of healthy cells to damage (selective stress resistance) - thus maximising relative dose intensity. Acute exercise can enhance blood flow to the tumours, oxygen delivery to them, thus enhance chemo delivery to the tumour and reduce tumour hypoxia. Combining both interventions e can down regulate cancer signalling pathways more so than either intervention alone. | Cycle ergometer, study diet (3 meals and 2 snacks per day from a selection) with a macronutrient composition designed to induce ketosis (80% fat, 10% protein, 10% cards). Will also be provided with a food diary to record dietary deviation and adverse symptoms. Given a handheld monitor to measure blood glucose and blood ketones. | One-time phone consultation with a registered dietitian and exercise physiologist providing general advice in line with the Canadian Cancer Society guidelines on nutrition and exercise, tailoring to the individual based on their REE data, habits, and experience. PLUS Given the study diet to follow for 72h prior to each scheduled start time of chemo (of only 48 hours if <7 days between infusions). Cycle ergometer during infusions: 5min warm up, 30-60minutues are target heart rate, 5-min cool down. Additional warm up or cool down may be performed. 2min breaks up to a maximum of 8 mins will be provided if required. Intravenous chemotherapy started in a chair and then participant moved to ergometer. | Registered dietitian and certified exercise physiologist | Individual, phone call and then in-person | In hospital | one-off phone call, study diet up to 6 times (before each cycle of chemo) 48 or 72 hours per diet time, cycling 6 times, during each infusion, typically lasting 40-70minutes: PA: sessions will be 5 min warm-up, 30-60minute at target heart rate, and 5 min cool down. 2 minute breaks permitted every 8 minutes. | Amount of food provided so it's 50% of each individual's daily energy expenditure. Differs depending on chemotherapy regimen. Between 21 to 28 day cycle. Intervention started 72hours before chemotherapy. When there is <7 days between infusions, participants follow the diet protocol for 48 hours prior to treatment. Meals are isocaloric with each representing 25% of daily energy expenditure and each snack representing 12.5%.  Exercise: Moderate intensity will be prescribed for the participants as 40 to 60% of age-predicted heart rate reserve, and dependant on abilities. Length of cycling adapted to length of chemotherapy. 2min breaks (up to 8 mins) allowed if required. | N/A | Participant diary describing any deviation from provided food, plus a handheld monitor to measure blood glucose and blood ketones after following the diet for 24, 48, 72h. | N/A |
| Meier-Girard, 2020^[55]^ | ERYT | Eurythmy may have positive effects on the autonomic nervous system - improved relaxation leads to better stress-coping strategies, better circadian rhythm, and improved QoL. | Not described | Cancer series 'O-E-M-L-I-B-D'. Active exercise therapy integrating cognitive, emotional, and volitional elements as well as sounds of language to express them in movements of the whole body. The inherent images of the sounds are performed by the therapist and recreated by the patient’s activity. One introductory session before the study. | Certified eurythmy therapists (who have completed a standardized 6 years of training, passed a federal examination, hold a federal diploma). | In-person small groups (1-4 people), with home practice. | Not reported. | 13 standardized therapy sessions of 45 min (once a week for 6 weeks and then once every second week) during the total intervention period of 20 weeks. PLUS Patients are asked to additionally train at home 15 min per day, 6 times a week. | Progression to more challenging movements based on patients' capabilities. | N/A | Compliance of home training reported in patient diary. | N/A |
| **Trial registries** | | | | | | | | | | | | |
| Ballinger, 2022^[56]^ | EMBody | Not described. | Heart rate monitors. | Virtual exercises including: cardiovascular exercise, resistance training, balance or stretching exercise. Goal of 40-60% of heart rate reserve (moderate intensity) during the sessions (as monitored by a wearable heart rate monitor). Based on their HR, RPE, and individual response during each sessions, trainers will follow an algorithm designed by the research team to progress or regress intensity level. They will also attend a class on creating and maintaining behaviour changes. | Exercise trainer | Virtual, live sessions (Zoom). | Remote (at home). | 3x a week for 16 weeks. Each session is 60 minutes. | Individualised based on heart rate, RPE, responses during the session. | N/A | Adherence with the intervention defined as the proportion of scheduled sessions attended by participants. | N/A |
| Borgquist, 2024^[66]^ | FEMA | Improving metabolic health and quality of life through physical activity in patients with metastatic breast cancer and overweight receiving endocrine-based treatment can optimize cancer care | Not reported | 12 week PA program 3x a week. High intensity training such as weight-lifting and cardiovascular training, and low-intensity training such as relaxation and stretching. | Supervised physiotherapist | In-person, supervised | Study clinic (Steno Diabetes Centre Aarhus) | 12 weeks, 3 times per week | Not reported | N/A | Adherence measured by attendance to programme (>=80%) | N/A |
| Chamberlin, 2012^[57]^ | Not described | Goal setting should increase PA, which in turn should demonstrate biologic and molecular differences compared to those who are not able to achieve that level of physical activity. The investigators expect that people who are more active will have longer to progression. | Educational materials on a DVD, logbook, pedometers. | Initial phone or in person consult to set goals, followed by weekly telephone (or in person) appointments (for the first month, followed by monthly) to inquire about symptoms, discuss meeting/not meeting goals and if not, how best to meet them. | Registered Nurse | Phone or in person | Remote or in person (not clear if hospital or home). | Weekly for the first month, then monthly for the rest of the trial. | Not reported | N/A | PA recorded on patient log and pedometers | N/A |
| Crane, 2024^[65]^ | FastER | Not reported | Participants will receive a Fitbit device to track their activity, and to provide heart rate monitoring during each session. Participants without access to Wi-Fi will be loaned cellular-enabled tablets to allow them to participate in telehealth sessions. Participants will also receive a notebook with information and support to encourage exercise. | 12-week supervised exercise programme delivered in-person or via telehealth; FitBit monitors heart rate during sessions | Trained health coach | In-person or telehealth | In-person sessions are not described, or telehealth delivered at home | 12 week programmes, sessions 3x a week, lasting 30-50 minutes. Total of 36 sessions | Length of session tailored according to participant tolerance, and based on fitness level and symptoms at study entry. | N/A | Not reported | N/A |
| Dal Molin, 2022^[64]^ | Intervention arm: WeCancer app + Smartwatch | Not reported | WeCancer app, Smartwatch (fitness tracker). | App may include: notifications with personalized advice (via chat), feedback to the patient in the comments made, guidance on physical and eating exercises, reception and support from the WeCancer multidisciplinary team. | Support provided by a navigator nurse, psychologist, nutritionist, physiotherapist. | Remotely delivered, individual | Remote (at home) | Recommended to use the app daily. | Personalised advice given in the chat. | N/A | Not reported | N/A |
| Devoto, 2014^[58]^ | AREA study | Not reported. | Treadmill, bike, rowing machine, weights. | The Exercise sessions include:  1. 10 minutes of warm-up using the large muscle groups in arms & legs on a treadmill, bike or rowing machine (mild paced and customized to individual capability)  2. 30 minutes of resistance exercise against own body weight or the weight of an object using arms and legs (customized to individual capability)  3. The last 10 minutes is for stretching exercises. | Under supervision of an experienced physiotherapist. | In-person. Individual or groups of no more than 4 participants. | Specialist Sports and Spinal Physiotherapy, Netherlands. | 1-hour sessions, twice a week, for 6 weeks. | Difficulty/intensity customised to individual capability. | N/A | Not reported | N/A |
| Harvie, 2015^[59]^ | B-AHEAD 3 | Many breast cancer patients are above an ideal weight, with high body fat and low muscle stores. Many patients gain fat and lose muscle during chemotherapy, and too much fat and low muscle stores can make chemotherapy less effective and increase its side effects. | Participants will be provided with demonstration online videos and leaflets on basic advice on healthy diet, chemotherapy side-effects related to diet and food hygiene, leaflet of their recommended exercise programme to use at home using an online physiotherapy tool, individual summary of key motivational issues. | Calorie-restricted diet and exercise programme consisting of resistance training. Individual session with dietitian, asked to follow the 5:2 diet (two restricted days consecutively, then a Mediterranean type diet for the remaining 5 days), including behavioural techniques to enhance dietary adherence. Individual session with physio/exercise professional to teach them the resistance exercise they will be asked to do. The exercise sessions include: brief warm up, 5-10 exercises for muscles in lower limbs, upper limbs, trunk. Then cool down. Exercises will have 3 progressive stages, with progression after 4-8 weeks, by increasing number of reps and sets and increasing resistance by using simple home light weights (e.g. bottle of water). | Trial dietitian, physiotherapist, or exercise specialist. | Shown exercises either by face to face or telephone appointment. Diet consultation face to face or by phone. | Exercise: either in person or remote. | The diet typically self-limits to 70–90 g protein and <60g fat/day and is limited in saturated and PUFA fat.  Resistance exercise: 3 sessions per week. Progression will be achieved with the same type of exercises by increasing the number of reps and sets and increasing resistance when exercising by using simple home light weights.  Diet: 45min appointment with dietitian. Exercise, one 45min appointment with physio/exercise specialist and then advised to do resistance training 3x per week. Phoned by study researchers every 3 weeks (20-30mins) to provide further advice and support and check progress. Face to face assessments every 6 months to ensure exercises are still safe and relevant. | The trial physiotherapist will adapt this standard  resistance programme for patients at baseline according to their metastatic site, co-morbidities and  current activity and energy levels | Not described | Fidelity of the interventions. Self -reported dietary intake (7-day food diary or diet history over the phone) and resistance exercise | N/A |
| Liu, 2020^[60]^ | Not described | Behavioural counselling increases PA | Information handbook, physical activity diary, FitBit. | Behavioural counselling to increase PA: Sessions will consist of discussing personal goals, barriers, enablers, pros and cons of physical activity and cognitive and behavioural strategies to aid in the process. Topics of discussion will be guided by the Transtheoretical Model of Behaviour Change. | Doctoral student, supervised by PhD supervisors. | In person and remote | First and final sessions in person at the university. All other sessions = remote (phone, video chat). | 12-week intervention. Weekly for first 6 weeks, fortnightly for final 6 weeks (10 sessions total). First and final sessions last 1.5-2 hours (to include assessment taking as well). All other sessions lasting 15-30 minutes. | Tailored according to each participant's context (stage of change, support needs, barriers). | N/A | Session completion, reasons for non-compliance/ withdrawal. | N/A |
| Lozano-Lozano, 2022^[61]^ | Overcome | not stated | Treadmill, ultrasound | Multi-modal individualised programme, supervised in person. This program will combine aerobic exercise in unloading using the TRAG (AlterG® Anti-Gravity Treadmill®), functional recovery of motor control with feedback ultrasound and occupational therapy. | MDT | Face to face | Not reported | 12 weeks, two sessions a week. | Individualised (not specified how). | N/A | Not reported | N/A |
| Reeves, 2022^[62]^ | Optimising care | Not described | Study workbook and logbook that outlines content delivered in sessions and the prescribed exercises, and helps participants keep track of the activities completed in between sessions. Also provided with resistance bands to assist with strength‐based exercises at home. | Access to maximum of 10 exercise and 10 diet one-to-one sessions. Exercise sessions: Given a weekly prescription. Focus on increasing aerobic-based and resistance-based exercise (body weight, resistance bands or weights) activities, with a goal of achieving greater than or equal to 150 minutes of activity per week moderate intensity or higher and including 2 sessions of resistance exercises. Likely that it will involve walking and stationary cycling as well as resistance exercises using bands. Diet: The Dietitian will work with participants to make sure that they are eating well (e.g., sufficient protein intake) with a focus on improving your overall diet quality and helping to manage symptoms. | Accredited exercise physiologist. Accredited practicing dietitian. | Remote - one-to-one video calls | Remote (at home) | 12-month programme. Initial sessions last 1 hour, subsequent ones last 30-45 minutes. Maximum of 10 sessions available for each component. | Yes, to the individual based on mets sites, treatment-related side-effects, baselines assessment of outcomes, and pre-screening questions. | N/A | Adherence to the sessions (Attendance, compliance to prescribed exercises) will be recorded during eHealth sessions. | N/A |
| Somayeh Nazari, 2023^[63]^ | Resistance training | NA | Not reported | Movements of the upper and lower body, based on American College of Sports Medicine. | Not described | In person | Hospital gym | For 8 weeks, 3 times a week, 4 movements of the upper body and 4 movements of the lower body. 8 to 12 repetitions in 2 to 3 sets. | At first, each person's 1RM is measured and the training intensity will be designed based on the person's initial power. | Not described | N/A | N/A |

N.B. LTF = Lost To Follow-up
